# Fast-Find: A novel computational approach to analyzing combinatorial motifs

**DOI:** 10.1186/1471-2105-7-1

**Published:** 2006-01-04

**Authors:** Micah Hamady, Erin Peden, Rob Knight, Ravinder Singh

**Affiliations:** 1 Department of Computer Science, University of Colorado, Boulder, CO 80309, USA; 2 Department of Molecular, Cellular and Developmental Biology, University of Colorado, Boulder, CO 80309, USA; 3 Department of Chemistry and Biochemistry, University of Colorado at Boulder, Boulder, CO 80309, USA

## Abstract

**Background:**

Many vital biological processes, including transcription and splicing, require a combination of short, degenerate sequence patterns, or motifs, adjacent to defined sequence features. Although these motifs occur frequently by chance, they only have biological meaning within a specific context. Identifying transcripts that contain meaningful combinations of patterns is thus an important problem, which existing tools address poorly.

**Results:**

Here we present a new approach, Fast-FIND (*Fast*-*F*ully *I*ndexed *N*ucleotide *D*atabase), that uses a relational database to support rapid indexed searches for arbitrary combinations of patterns defined either by sequence or composition. Fast-FIND is easy to implement, takes less than a second to search the entire *Drosophila *genome sequence for arbitrary patterns adjacent to sites of alternative polyadenylation, and is sufficiently fast to allow sensitivity analysis on the patterns. We have applied this approach to identify transcripts that contain combinations of sequence motifs for RNA-binding proteins that may regulate alternative polyadenylation.

**Conclusion:**

Fast-FIND provides an efficient way to identify transcripts that are potentially regulated via alternative polyadenylation. We have used it to generate hypotheses about interactions between specific polyadenylation factors, which we will test experimentally.

## Background

DNA- and RNA-binding proteins are essential for the regulation of gene expression at many levels. They control many biological processes in all organisms by altering gene expression at the levels of transcription, pre-mRNA splicing, mRNA export, stability, localization, and translation. Although some proteins bind specific sequences, others bind short or degenerate patterns, also called motifs, that occur frequently in the genome by chance. These patterns can even be defined by base composition rather than by an exact sequence.

Proteins that bind frequently-occurring sites cannot individually be highly specific, but such proteins can achieve specificity by cooperation in complexes clustered near regulatory sequences. This combinatorial control is the rule rather than the exception in higher eukaryotes for critical processes including transcription [1] and splicing [2], and has also been observed in bacterial transcription [3]. Building up regulatory complexes in this way, rather than using individual gene- or transcript-specific factors, confers many advantages. These advantages include tissue-specific fine-tuning of biological responses through interactions with different combinations of proteins, increased evolutionary stability by mitigating deleterious effects of changes in an individual pattern, and repeated recognition of sequences by dynamic multi-protein complexes such as the spliceosome [4].

Unfortunately, this combinatorial flexibility substantially complicates computational searches for patterns involved in function, because most occurrences of most patterns are not biologically meaningful. Although there are many well-established approaches for defining and searching for patterns in strings, these techniques typically either require a linear scan of the sequence or cannot deal with compositionally-defined patterns. Examples of the former type include regular expressions (reviewed in [5]) and weight matrices (reviewed in [6]); examples of the latter include suffix trees and suffix arrays (reviewed in [7]). Because the patterns for nucleic acid binding proteins are often poorly defined, it is critical to avoid a linear-time search, and to be able to conveniently locate multiple patterns near regions of biological interest. In this paper, we present Fast-FIND (*Fast*-*F*ully *I*ndexed *N*ucleotide *D*atabase), a new algorithm that addresses this class of problem by indexing sequences in a relational database.

As an example of the application of Fast-FIND to the study of combinatorial regulation, we searched for patterns potentially involved in alternative polyadenylation. Polyadenylation, or addition of a poly(A) tail at the 3' end of a cleaved mRNA, is required for the synthesis of almost all mRNAs in higher eukaryotes [8,9]. The polyadenylation machinery recognizes a combination of two patterns: a conserved AAUAAA consensus polyadenylation signal located between 10 and 30 nucleotides upstream of the cleavage site, and a relatively flexible GU-rich enhancer element located between 20 and 40 nucleotides downstream of the cleavage site [10,11].

Alternative polyadenylation, in which the transcripts from a single gene have alternative 3' ends, is an important but poorly-studied process. Although both tissue-specific and disease-specific differences in alternative polyadenylation patterns have been reported [9,10,12-14], and many transcripts in different organisms have alternative 3' ends (50% in human, 31% in mouse, 28% in rat and 25% in *Arabidopsis*) [15], little is known about how this important process of gene regulation occurs. We chose to study alternative polyadenylation in *Drosophila *because this model organism can be conveniently manipulated by genetic techniques to confirm predictions from genome-wide sequence analysis.

## Implementation

### Databases and programming

We downloaded sequences for *Drosophila *complementary DNAs (cDNAs) [16] and expressed sequence tags (ESTs) [17] in FASTA format. All source code for Fast-FIND, written in Python, is available from the authors upon request.

We implemented Fast-FIND in a relational database because relational database management systems (RDBMS) automatically provide solutions to many problems that would need to be considered if using custom data structures and code to support searches. These solutions include a standard, declarative language for performing queries (SQL), efficient indexing mechanisms and caching strategies, support for concurrent access by multiple users, and scalability to multiple processors and/or distributed systems [18].

Fast-FIND consists of two phases: a one-time, O(N) indexing phase, which generates and populates the database tables, and an O(log N) search phase, in which arbitrary queries can be evaluated. We now describe these phases in detail.

### Fast-FIND indexing phase

#### Overview

During the Fast-FIND indexing phase, we analyze each sequence using overlapping 32-base sliding windows. We pack the bases in each window into a bitvector (an array of 1's and 0's, which allows us to perform efficient comparisons of strings by treating them as numbers) and store them in the database. We also use overlapping, variable-sized sliding windows to calculate the composition of each 3- to 20-base substring within the sequence. This indexing produces two ancillary tables. One table contains the bitvector and position of each 32 base window, while the other contains a window size, a pointer to the window composition, and the window position in the longer sequence. The bitvector table is used for exact matching, and the composition table is used for compositionally defined patterns.

#### Sequence indexing

**Figure 1:**
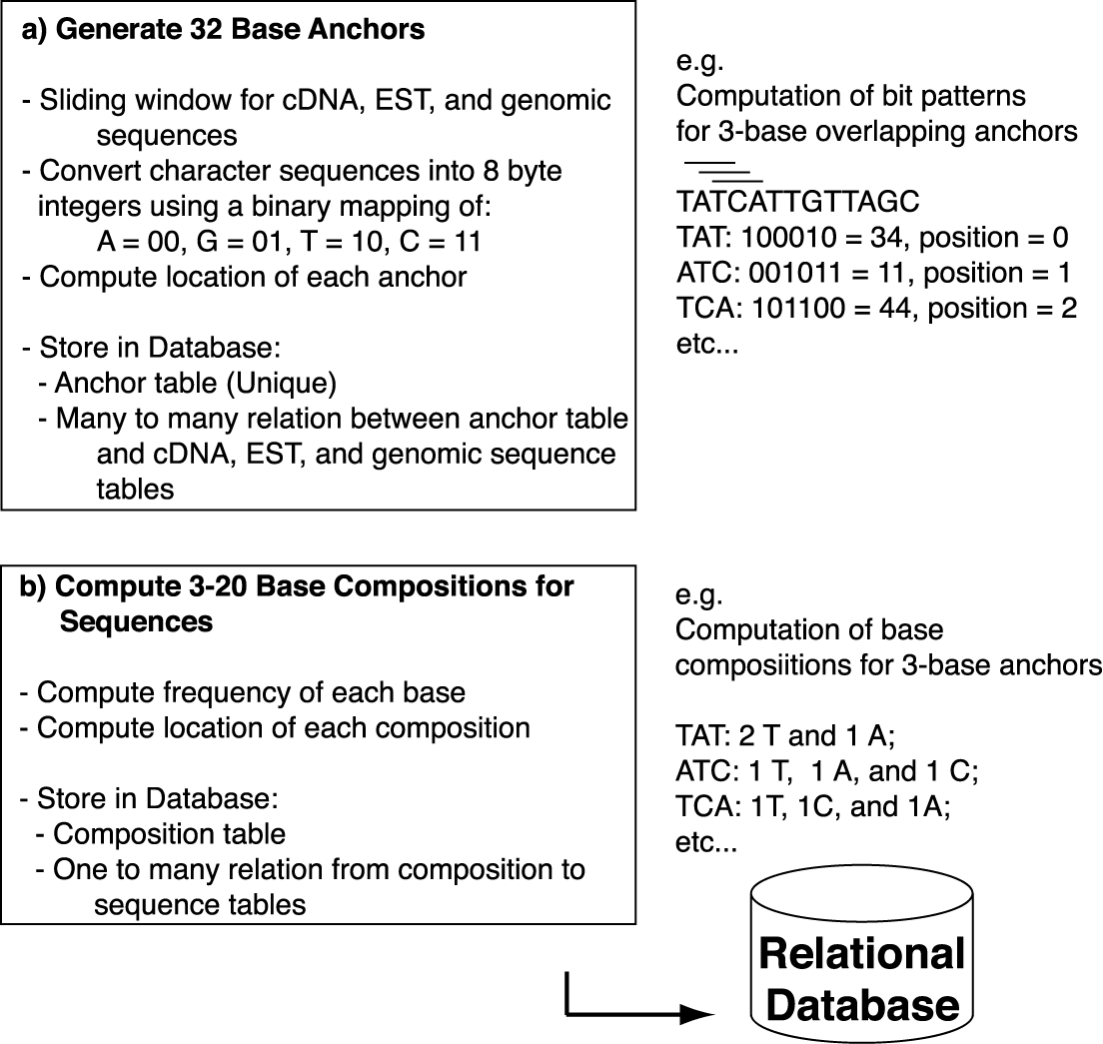
**Fast-FIND indexing phase**. Summary of the strategies for generating and storing (a) bitvectors for 32-base windows and (b) compositions of 3–20 base windows for each sequence.

For each unambiguous position *N *in the sequence we compute and store an 8-byte integer from the 32 base window, [N:N+32] for exact matching (Figure 1a), which we associate with the identifier of the matching sequence and the position where the match occurred. To save both computation time and space, we use a bitvector encoding scheme that packs non-degenerate sequences into 2 bits per base: the first bit indicates purines (AG) or pyrimidines (UC), and the second indicates weak (AU) or strong (GC) H-bonding for base pairing. This type of 2-bit encoding has been widely used elsewhere (see for example, [19]). Each base maps to a specific bit pattern: A maps to 00, G to 01, U/T to 10, and C to 11. We excluded the few records in our data set that contained degenerate bases, accounting for less than 0.01% of the data.

#### Composition indexing

For each unambiguous position *N *in the sequence we compute and store the base composition of all 3- to 20-base windows [N:N+y], where y is the length of the window (Figure 1b). We thus calculate and store the location and composition of all sliding windows from 3 to 20 bases in length within each sequence; all biologically relevant protein-binding sites in RNA that we are aware of fall within this range.

### Fast-FIND search phase

#### Overview

Fast-FIND supports composition and exact searches by numerical range operations on the composition and sequence indexes respectively. It supports inexact searches (searches for a pattern defined using IUPAC degenerate symbols) using application logic rather than within the database.

#### Composition searches

To find a pattern of specified length and composition, we use range operations to look up all possible counts of each of the four bases that could match the pattern, and join these compositions to the table containing the locations of the windows that match them. This design allows us to rapidly locate arbitrarily degenerate patterns with a specified range of compositions.

#### Exact pattern searches

**Figure 2:**
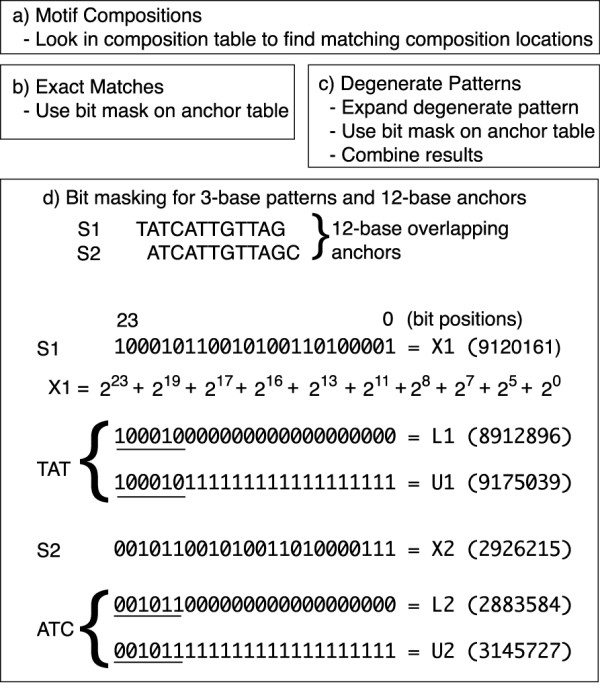
**Searches for different types of patterns**. Searching for sequence patterns based on (a) base composition, (b) exact matches, and (c) degenerate base patterns. (d) A simplified example of the bit masking approach for 3-base patterns and 12-base windows. Calculate the integer X1 for the string S1 as the sum of 2^bit-pos^, where bit-pos refers to bit positions (0 to 23) for each bit set to 1. Each bitvector is followed by its corresponding decimal value (in parentheses). Similarly, calculate integer values for the overlapping string S2 and for the upper (U1 and U2) and lower (L1 and L2) bounds for two search patterns (TAT and ATC). The bit patterns for windows S1 and S2 are shown using the notation for bases in Figure 1a. The bit patterns for the search patterns, TAT and ATC, are indicated by an underline, and the remaining positions are masked with a value of either 0 or 1 for the lower and upper integer limits (as shown for S1), respectively. X1 is between L1 and U1, but not between L2 and U2. Similarly, X2 is between L2 and U2, but not between L1 and U1. This example demonstrates that S1 begins with TAT but not ATC, and S2 begins with ATC but not TAT.

To find exact patterns up to 32 bases (for a simplified example, see Figure 2), we search the sequence index for windows that contain the pattern as a prefix. Instead of storing all window sizes up to 32 bases (as for the composition index), we use bit masking to simulate the effect of storing smaller windows. Because we store each window as an 8-byte integer (64 bits for the 32 bases), we can perform searches by finding all windows where the S bits in a given pattern (where S is a positive integer ≤ 64) exactly matched the first T bits of the window. To examine the first T bits of a 64-bit window we need to mask out the remaining (64-T) bits. Since 2^n > (2^n-1 + 2^n-2 + ... + 2^0), we can generate a mask of all 0's for bit positions < 64-T-1 for the lower bound, L, and a mask of all 1's for bit positions < 64-T-1 for the upper bound, U. Thus, the first T bits in an integer between L and U will match the S bits of the given pattern. However, if there is a position Y in the first T bits of a window that differs from the corresponding position in the S bits of the given pattern, the computed search integer for the given pattern will be 2^((64-T-1) +Y) > U or 2^((64-T-1) +Y) < L. Thus, by simply searching for a bounded range of integers, we can immediately find the location of any S/2 base pattern in the sequences.

#### Degenerate pattern searches

We support searches using patterns containing any of the IUPAC degenerate bases using two methods, depending on the level of degeneracy. In the first method, we generate all possible sequences that match the pattern, and then combining the search results of exact matching from each sequence. Although this technique is exponential (O(4^N^)) in the number of unspecified positions, five degenerate bases (1024 combinations) are sufficient to identify essentially all well-characterized binding sites for RNA-binding proteins [20,21] and imposes little computational burden in practice. In the second method, we use a different encoding scheme in which each position is represented by four bits, indicating presence/absence of each base in the degenerate symbol, and perform masked searches. This latter method requires a linear table scan, making it O(N) in the number of indexed windows, but is useful for highly degenerate searches and for searches against sequences containing ambiguous bases.

### Applying Fast-FIND to alternative polyadenylation in *Drosophila*

#### Overview

**Figure 3:**
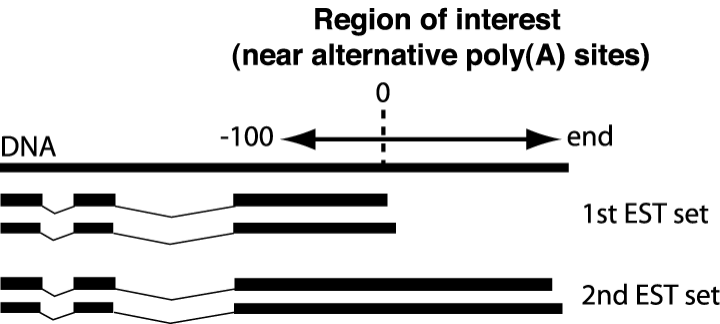
**Identifying regions of interest through cDNA/EST matches**. The region of interest located between 100 nucleotides upstream of the 3' end of the first EST set through the 3' end of the second EST set was indexed.

To apply Fast-FIND to the study of alternative polyadenylation, we needed to identify alternatively polyadenylated transcripts. Rather than using annotated polyadenylation sites, which are unreliable, we identified alternative polyadenylation by fully indexing the *Drosophila *cDNA and EST libraries downloaded from NCBI and identifying cDNAs that contained both 3' and internal matches to ESTs annotated as "3prime" or "complete". We then defined the region of interest for alternative polyadenylation as the region from 100 bases upstream of the first internal match to a cDNA through the end of the sequence (Figure 3). Finally, we constructed search tables containing only this region of interest to reduce the table sizes, increasing performance.

#### Mapping cDNAs and ESTs onto the genome

We used BLAT [22] to map cDNAs and ESTs onto the genome, which also provided information about which ESTs matched which cDNAs (because both matched the same positions on the genome).

#### Identifying candidates for alternative polyadenylation

We defined cDNAs with alternative 3' ends as candidates for alternative polyadenylation. These candidates were identified by ensuring that at least two sets of ESTs annotated as "3prime" or "complete" matched somewhere on the same cDNA. The 'region of interest' for alternative polyadenylation is defined as the region within a candidate cDNA sequence that begins 100 nucleotides upstream of the end of the first EST set through the end of the cDNA (Figure 3). Sequences within this region were further indexed to support pattern searches as described above.

#### Properties of the alternative polyadenylation database

The relational database took ~2 hours to build on a 2.4 GHz Pentium 4 Dell Optiplex, a one-time investment because incremental updates can be used to add new data as necessary, and required ~2 GB (for both tables and indexes) of disk space. For comparison, the original EST library was 165 MB and the original cDNA library was 20 MB. From the matches between *Drosophila *cDNAs and ESTs, we determined the subset of cDNAs that showed evidence of potential alternative polyadenylation. ~12% of the EST sequences matched the 3' ends of the corresponding mRNAs, the remainder matching the 5' ends. We identified ~470 candidate cDNAs for further indexing. These restrictions minimize potential concerns about multi-gene families and avoid artifacts from contaminants in the EST libraries [23]. As a positive control for the method, *vimar *shows two alternatively polyadenylated transcripts for which the 3'ends in the database are supported by experimental analysis [24]. As expected, queries for sequences from only the region of interest specifically identify this candidate using Fast-FIND.

### Confirming the significance of motif co-occurrence

To test whether pairs of binding sites were correlated in abundance, we used the G test to determine whether transcripts that contained one site were more likely to contain the other. The G test is a test for association similar to the familiar chi-squared test widely used in genetics, but is more accurate for small sample sizes [25]. To confirm the G test results, we performed Monte Carlo simulations of the sequences using three Markov models: (a) first-order Markov model using the full set of regions of interest as a training set; (b) fifth-order Markov model using the full set of regions of interest as a training set; and (c) permuting the nucleotides in each sequence independently. The first two of these models preserve the single-base and five-base 'word' frequencies respectively, while the third model accounts for compositional heterogeneity between the sequences. For each model, we made 1000 random sets of sequences of the same length as the actual sequences, recalculated the G statistic using each random set, and compared the value of the G statistic in the actual set to that in each random set. The empirical P-value was estimated as the fraction of random sets with lower G scores than the actual set. This analysis corrects for any error due to biases in the length or composition of the alternatively polyadenylated transcripts. As an additional control, we verified that the counts and locations of motifs identified by Fast-FIND were identical to those found by using Python's built-in string-matching facility.

### Web interface

We have set up a web form allowing searches of arbitrary combinatorial patterns against the portions of the *Drosophila *cDNAs relevant for alternative end formation and retrieval of relevant sequences at [26].

## Results

### Performance characteristics for arbitrary pattern searches

We tested the effects of several variables on the time taken to search for patterns in the database within regions of interest for alternative polyadenylation. The time taken to perform an exact search was independent of the size of the pattern; it took <0.02 seconds for patterns expanding to 1 to 64 sequences, containing 6, 10, 15, or 20 nucleotides and 0, 1, 2, or 3 degenerate nucleotides. In addition, for a compositionally defined pattern (T ≥ 15, G ≤ 2, (C + A) = 0, length = 17), there were 154 possible matching sequences: this search identified 5 cDNAs, and took 0.02 seconds. Thus, Fast-FIND provides an efficient tool to search for arbitrary user defined pattern(s) (Table 1).

### Identification of cDNAs containing binding sites for RNA-binding proteins

In eukaryotes, binding sites and biologically relevant targets are known for very few of the several hundred known RNA-binding proteins [21,27]. We tested whether several well-characterized RNA-binding proteins were significantly associated with one another near alternative polyadenylation sites. For this analysis, we used several RNA-binding proteins for which binding sites have been identified by iterative selection amplification or biochemically: Sex-lethal (SXL) [28,29], RBP1 [30], P-element somatic inhibitor (PSI) [31], Rbp9 [32], and hnRNP H/H'/F family [33]. The binding sites for SXL and CstF64 are both defined by base composition rather than by exact sequence [28,29,34], highlighting the necessity for both composition- and sequence-based searches. The similarities between the U-rich binding sites for RBP1 (and SXL) and a subset of the GU-rich polyadenylation enhancers or CstF64 binding sites suggested to us that they might affect alternative polyadenylation [9,12,13]. SXL regulates specific 5' and 3' splice sites by blocking the binding of a specific splicing factor, U2AF, [35]. Thus it might similarly regulate alternative polyadenylation by binding to and blocking certain GU-rich polyadenylation enhancers, thus activating an alternative polyadenylation site.

#### Searching for SXL-regulated polyadenylation: a natural compositionally-defined site

If an RNA-binding protein such as SXL affects alternative polyadenylation, we would expect to find its consensus binding site close to the first 3' end of alternatively polyadenylated transcripts from the same gene. We used Fast-FIND to search for cDNAs meeting these criteria.

**Figure 4:**
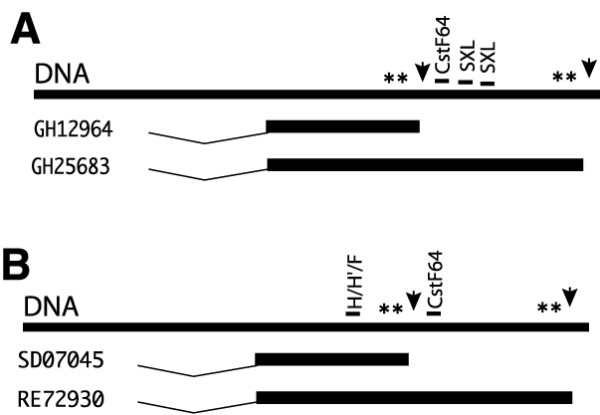
**A schematic of potential candidates for alternative polyadenylation**. Arrowheads show 3' ends, asterisks show the consensus polyadenylation signal, and potential SXL, hnRNP H/H'/F, and CstF64 sites are indicated.

Table 1 shows the number of cDNAs that had both multiple 3' ends and SXL-binding sites. We used a range of different pattern definitions for the SXL site, ranging from 8 to 17 nucleotides, which differ in binding affinity for SXL. Five cDNAs both showed support for alternative polyadenylation and contained the pattern that most closely approximates the natural SXL-binding site found in *tra*, a known SXL target (T ≥ 15, G ≤ 2, (C + A) = 0, length = 17) [36]. Thus, we have identified cDNAs containing potential SXL-binding sites that are promising candidates for regulation via alternative polyadenylation. One of these candidates, which we independently confirmed by a separate analysis using BLAST to assign alternatively polyadenylated regions and regular expressions to find a subset of the SXL patterns, is displayed in Figure 4A.

#### Searching for combinatorial patterns of degenerate binding sites

In natural situations, genes are typically regulated by combinations of binding sites rather than by single binding sites [1-4]. We tested whether the binding sites for several well-characterized RNA-binding proteins occurred in the same alternative polyadenylation region more frequently than chance would predict. Specifically, for each pair of binding sites, we used the G test to determine whether transcripts that contained one site were more likely to contain the other. The G test is a test for association similar to the familiar chi-squared test widely used in genetics, but is more accurate for small sample sizes [25].

Although the initial G tests indicated that the RNA-binding patterns for CstF64 and the hnRNP H/H'/F family members are significantly associated in the alternatively polyadenylated regions of *Drosophila *cDNAs (Table 1), the Monte Carlo analysis did not confirm the significance of these associations. In contrast, associations between SXL and CstF64, SXL and the hnRNP core, and PSI and the hnRNP core were marginally statistically significant in the G test but were highly significant (P < 0.005) in all three Monte Carlo simulations, even when heterogeneity in composition and overlap between the sites were accounted for. One cDNA that provides an example of potential regulation by the hnRNP H/H'/F family of RNA-binding proteins is shown in Figure 4B.

## Discussion

We have used Fast-FIND to identify genes that are promising candidates for regulation by alternative polyadenylation. We have demonstrated that Fast-FIND can be used to rapidly search for both individual and multiple patterns within defined regions of sequence that are biologically important, and that the queries are sufficiently fast to support the analyses of many different variations on pattern, which is important when knowledge about the pattern is limited.

The two proteins that we found to be associated near alternative polyadenylation sites, CstF64 and the hnRNP H/H'/F, have previously been linked to polyadenylation site choice [37-40]. They are not known to interact in the cell, and, in this context, it is interesting that their sites are not significantly co-located, although each is significantly co-located with other RNA-binding proteins, including SXL. The other proteins we examined have not been implicated in polyadenylation regulation, but may act as cofactors with CstF64 or hnRNP H/H'/F. We have thus used our new tool to generate novel hypotheses about specific biological interactions that we now plan to test experimentally. Our identification of alternatively polyadenylated mRNAs that contain specific combinations of binding sites thus opens up possibilities for new studies of both gene function and the molecular mechanisms by which polyadenylation site choice regulates genes. Most importantly, because each of the individual short, degenerate binding sites occurs frequently, identification of such candidates would not have been possible without using associations between the different sites.

Fast-FIND offers several advantages over other solutions to the pattern-matching problem. First, except for the one-time investment of computational resources during the indexing phase (database updates are incremental, requiring minimal additional CPU time), the indexed approach eliminates the linear scan for every new pattern. Consequently, many different combinations of search patterns can be evaluated until an experimentally manageable number of potential cDNAs is identified for further analysis. Second, Fast-FIND allows searches for combinatorial patterns. This capability is particularly important because most eukaryotic genes are regulated by a combination of multiple sequence patterns rather than by a single pattern. Third, Fast-FIND is uniquely suited to finding short patterns defined by composition. Because each window has only one composition, and because the compositions are inherently ordered according to the number of each base they contain, we can store the composition for each window in the database and then search for all windows within any arbitrary range of compositions rapidly. In contrast, it is impossible to precalculate matches for all possible regular expressions or for all possible weight matrices, making it difficult to avoid a new O(N) linear scan through the sequence when searching for a new pattern. Finally, Fast-FIND is easily implemented using standard database software, making it easily accessible to a broad audience.

The indexing approach used in Fast-FIND is not appropriate for all applications. The database tables take up much more space than the original sequences, limiting its use for extremely large data sets. Generalized suffix trees and suffix arrays provide better performance in a smaller space in cases where the entire data structure can fit into main memory and concurrent access by multiple users is not required. Weight matrices are more suitable for highly degenerate sequences, where the O(N) in the number of possible matching patterns can become larger than a linear scan that is O(N) in the length of the sequence to be searched. The specific indexing scheme we used for this analysis does not handle degenerate bases, although we provide this capability by using a different bitvector scheme, which degrades the search performance to a linear scan (data not shown). However, even taking these limitations into account, Fast-FIND is useful for a wide range of biologically important problems.

## Conclusion

Fast-FIND provides a versatile tool for searching for exact and compositionally defined search patterns. We have applied this approach to analyzing the role of RNA-binding proteins with known binding sites in regulating alternative polyadenylation in *Drosophila*, and have found several mRNAs that are plausible targets for known RNA-binding proteins. Although the database we created is *Drosophila*-specific, the general approach described here can easily be extended to the analysis of combinatorial regulation in other species and in other contexts, including the control of transcription, splicing, mRNA stability, and translation.

## Availability and requirements

The Fast-FIND interface is available at . The software is available under the GPL by request to the authors.

## Abbreviations

Fast-Fully Indexed Nucleotide Database, Fast-FIND; complementary DNAs, cDNAs; expressed sequence tags, ESTs; relational database management systems, RDBMS; International Union of Pure and Applied Chemistry, IUPAC; Sex-lethal, SXL; P-element somatic inhibitor, PSI; heterogeneous nuclear ribonuclear protein, hnRNP.

## Authors' contributions

MH implemented Fast-FIND and improved the algorithm; EP curated the database and independently analyzed the presence of alternative polyadenylation sites; RK designed the Fast-FIND algorithm and performed statistical analysis; and RS proposed the biological problem, designed variants of the SXL-binding site, assisted in EP's analysis, and drafted the manuscript. All authors read and approved the final manuscript.

## Figures and Tables

**Table 1:** Candidates with desired binding sites adjacent to alternative polyadenylation sites. Identification of cDNAs with potential alternative 3' ends and various patterns – base composition, degenerate, and combinatorial patterns – located between 100 nucleotides upstream of the 3' end of the first EST set through the 3' end of the second EST set. # and ** cDNAs were used as examples for the alignment shown in Figure 4.

Number of cDNAs with potential alternative 3' ends and search patterns		
Search pattern	Number of Patterns	Number of cDNAs

A. Base composition		
CstF64; U> = 4, G< = 4, A+C = 0 ;length = 8	163	276
SXL; U> = 15, G< = 2, A+C = 0 ;length = 17	154	5
SXL1; U> = 8, G+A+C = 0 ;length = 8	1	27
SXL2; U> = 10, G< = 2, A+C = 0 ;length = 12	79	25
		
B. Degenerate motifs		
hnRNP F/H/H' (core); GGGA	1	232
hnRNP F/H/H'; GGGGA	1	78
Rbp1; DCADCUUA	9	47
PSI; RCYYCUURYRC	12	8
Rbp9; UUUNUUUU	4	111
		
C. Combinatorial motifs		
CstF64 + SXL	25,102	5^#^
CstF64 + hnRNP F/H/H' (core)	163	178*
CstF64 + hnRNP F/H/H'	163	59**
SXL + hnRNP F/H/H' (core)	154	4***
PSI + hnRNP F/H/H' (core)	12	8***

# Since both SXL and CstF64 sites are GU rich, these motifs are not expected to be statistically independent. However, all three Monte Carlo analyses showed that the association was significant (P < 0.001) even when accounting for composition, indicating that SXL sites are more likely to also be CstF64 sites than chance predicts.

* and ** Associations are statistically significant by the G test:

(* G = 69.8, P = 3.3 × 10^-17^, df = 1; and **G = 11.6, P = 0.00033, df = 1). However, these associations were not significant in the Monte Carlo.

*** Associations not individually significant by the G test, but significant (<0.01) in all three Monte Carlo tests.

Associations of various other combinations of SXL, Rbp1, PSI, and Rbp9 motifs in cDNAs are not statistically significant.

## References

[B1] Levine M, Tjian R (2003). Transcription regulation and animal diversity. Nature.

[B2] Smith CW, Valcarcel J (2000). Alternative pre-mRNA splicing: the logic of combinatorial control. Trends Biochem Sci.

[B3] Buchler NE, Gerland U, Hwa T (2003). On schemes of combinatorial transcription logic. Proc Natl Acad Sci U S A.

[B4] Singh R, Valcarcel J (2005). Building specificity with nonspecific RNA-binding proteins. Nat Struct Mol Biol.

[B5] Friedl JEF (1997). Mastering Regular Expressions.

[B6] Durbin R, Eddy S, Krogh A, Mitchison G (1998). Biological Sequence Analysis.

[B7] Gusfield D (1997). Algorithms on strings, trees and sequences: computer science and computational biology.

[B8] Dominski Z, Marzluff WF (1999). Formation of the 3' end of histone mRNA. Gene.

[B9] Hirose Y, Manley JL (2000). RNA polymerase II and the integration of nuclear events. Genes Dev.

[B10] Colgan DF, Manley JL (1997). Mechanism and regulation of mRNA polyadenylation. Genes Dev.

[B11] Manley JL, Takagaki Y (1996). The end of the message--another link between yeast and mammals. Science.

[B12] MacDonald CC, Redondo JL (2002). Reexamining the polyadenylation signal: were we wrong about AAUAAA?. Mol Cell Endocrinol.

[B13] Zhao J, Hyman L, Moore C (1999). Formation of mRNA 3' ends in eukaryotes: mechanism, regulation, and interrelationships with other steps in mRNA synthesis. Microbiol Mol Biol Rev.

[B14] Bentley D (1999). Coupling RNA polymerase II transcription with pre-mRNA processing. Curr Opin Cell Biol.

[B15] Yan J, Marr TG (2005). Computational analysis of 3'-ends of ESTs shows four classes of alternative polyadenylation in human, mouse, and rat. Genome Res.

[B16] database DNA [http://www.fruitfly.org/sequence/sequence_db/na_cDNA.dros].

[B17] database EST [http://www.fruitfly.org/sequence/sequence_db/na_EST.dros].

[B18] Lewis PM, Kifer M, Bernstein AJ (2002). Database and Transaction Processing.

[B19] Chen X, Kwong S, Li M (1999). A Compression Algorithm for DNA Sequences and Its Applications in Genome Comparison. Genome Inform Ser Workshop Genome Inform.

[B20] Tacke R, Manley JL (1999). Determinants of SR protein specificity. Curr Opin Cell Biol.

[B21] Varani G, Nagai K (1998). RNA recognition by RNP proteins during RNA processing. Annu Rev Biophys Biomol Struct.

[B22] Kent WJ (2002). BLAT--the BLAST-like alignment tool. Genome Res.

[B23] Beaudoing E, Freier S, Wyatt JR, Claverie JM, Gautheret D (2000). Patterns of variant polyadenylation signal usage in human genes. Genome Res.

[B24] Lo PC, Frasch M (1998). bagpipe-Dependent expression of vimar, a novel Armadillo-repeats gene, in Drosophila visceral mesoderm. Mech Dev.

[B25] Sokal RR, Rohlf FJ (1995). Biometry: the principles and practice of statistics in biological research.

[B26] Fast-FIND [http://bmf.colorado.edu/fastfind/].

[B27] Burd CG, Dreyfuss G (1994). Conserved structures and diversity of functions of RNA-binding proteins. Science.

[B28] Singh R, Valcarcel J, Green MR (1995). Distinct binding specificities and functions of higher eukaryotic polypyrimidine tract-binding proteins. Science.

[B29] Sakashita E, Sakamoto H (1994). Characterization of RNA binding specificity of the Drosophila sex- lethal protein by in vitro ligand selection. Nucleic Acids Res.

[B30] Heinrichs V, Baker BS (1995). The Drosophila SR protein RBP1 contributes to the regulation of doublesex alternative splicing by recognizing RBP1 RNA target sequences. Embo J.

[B31] Amarasinghe AK, MacDiarmid R, Adams MD, Rio DC (2001). An in vitro-selected RNA-binding site for the KH domain protein PSI acts as a splicing inhibitor element. Rna.

[B32] Park SJ, Yang ES, Kim-Ha J, Kim YJ (1998). Down regulation of extramacrochaetae mRNA by a Drosophila neural RNA binding protein Rbp9 which is homologous to human Hu proteins. Nucleic Acids Res.

[B33] Caputi M, Zahler AM (2001). Determination of the RNA binding specificity of the heterogeneous nuclear ribonucleoprotein (hnRNP) H/H'/F/2H9 family. J Biol Chem.

[B34] Takagaki Y, Manley JL (1997). RNA recognition by the human polyadenylation factor CstF. Mol Cell Biol.

[B35] Valcarcel J, Singh R, Zamore PD, Green MR (1993). The protein Sex-lethal antagonizes the splicing factor U2AF to regulate alternative splicing of transformer pre-mRNA. Nature.

[B36] Sosnowski BA, Belote JM, McKeown M (1989). Sex-specific alternative splicing of RNA from the transformer gene results from sequence-dependent splice site blockage. Cell.

[B37] Takagaki Y, Seipelt RL, Peterson ML, Manley JL (1996). The polyadenylation factor CstF-64 regulates alternative processing of IgM heavy chain pre-mRNA during B cell differentiation. Cell.

[B38] Takagaki Y, Manley JL (1998). Levels of polyadenylation factor CstF-64 control IgM heavy chain mRNA accumulation and other events associated with B cell differentiation. Mol Cell.

[B39] Veraldi KL, Arhin GK, Martincic K, Chung-Ganster LH, Wilusz J, Milcarek C (2001). hnRNP F influences binding of a 64-kilodalton subunit of cleavage stimulation factor to mRNA precursors in mouse B cells. Mol Cell Biol.

[B40] Proudfoot NJ, Furger A, Dye MJ (2002). Integrating mRNA processing with transcription. Cell.

